# Perception about child vaccination and its determinants among caregivers in rural Northeastern Ethiopia

**DOI:** 10.1007/s43999-026-00097-1

**Published:** 2026-08-12

**Authors:** Seid Assega Beshir, Mahteme Haile Workineh, Birhanu Demeke Workineh, Mesfin Haile Kahissay

**Affiliations:** 1 Department of Pharmacy, College of Medicine and Health Sciences, Wollo University, Dessie, Ethiopia; 2 School of Pharmacy, College of Health Sciences, Addis Ababa University, Addis Ababa, Ethiopia; 3 House of People Representative of FDRE, Addis Ababa, Ethiopia

**Keywords:** Vaccination, Community perception, Tehuledere Woreda, Children 12–36 months, Ethiopia

## Abstract

**Background:**

Despite increased global immunization, many children in developing countries remain unvaccinated. Effective communication with parents is crucial to overcoming barriers and improving vaccination coverage.

**Objective:**

To examine caregivers’ perception towards child vaccination and identify factors associated with incomplete vaccination status (as an indicator of vaccine hesitancy) among children 12–36 months of age in Tehuledere Woreda, South Wollo Zone, North East Ethiopia.

**Methods and materials:**

An explanatory sequential mixed method was used. A community-based cross-sectional study was conducted from September 1 to November 30, 2019 with a sampling technique. A total of eight hundred twenty-four mothers/caregivers of children aged 12–36 months were systematically selected and interviewed using a structured questionnaire. Data were analyzed using SPSS version 20. Logistic regression and content analysis were performed.

**Results:**

A total of 596 (72.3%) of the children were completely vaccinated. Widowed mothers (AOR = 7.3, 95% CI = 2.2,24), home delivery practice (AOR = 4.6, 95% CI = 1.8,10), long walking time to health facility (AOR = 9.5, 95% CI = 3.2,27), use of community as vaccine information source (AOR = 4.2, 95% CI = 1.7,9.6), occurrence of child death in the family (AOR = 2.6, 95% CI = 1.2,6.8) and mothers’ health seeking behavior (AOR = 2.5, 95% CI = 1.1,5.4) were found to be associated with incomplete vaccination.

**Conclusion and recommendations:**

Acceptance and refusal of child vaccination reflected factors including perceived benefits of vaccine and social environment. Encouraging mothers to attend proper antenatal care and to utilize family planning programs is recommended to improve vaccination status of children in the rural communities. Community awareness programs also should be scaled up by incentivizing community health workers.

**Supplementary Information:**

The online version contains supplementary material available at 10.1007/s43999-026-00097-1.

## Introduction

Immunization is a cornerstone of public health, substantially reducing the incidence and mortality associated with infectious diseases and positively impacting social phenomena and community well-being. However, the global public health community faces a significant challenge known as vaccine hesitancy, particularly evident in Western countries, where concerns about vaccine safety, efficacy, and necessity persist [1]. This hesitancy has been acknowledged by the World Health Organization (WHO) as a ‘growing challenge for immunization programs,’ ranking among the top 10 threats to global health [2–4].

The emergence of vaccine-preventable diseases (VPDs), including measles, poliomyelitis, diphtheria, and pertussis, in developed regions has been linked to under-vaccinated or non-vaccinated communities [5, 6]. Under-vaccination is influenced by diverse factors, and even vaccinated individuals express doubts and concerns [3]. Decision-making regarding vaccination involves a spectrum from complete refusal to confident acceptance [7], influenced by cognitive, psychosocial, and political factors within the scientific, cultural, and media environments [8].

Notably, reports suggest that vaccination coverage in many African countries, including Ethiopia, has plateaued at suboptimal levels [9]. Factors contributing to this plateau include limited access to vaccination services, inadequate resources, and vaccine hesitancy [10, 11]. The WHO reported a steady increase in the number of countries experiencing vaccine hesitancy since 2014, with only a few reporting no hesitancy in 2015–2017 [12]. In response, the WHO declared vaccine hesitancy as one of the top ten threats to global health in 2019.

In the context of these challenges, the Amhara Regional Health Bureau has aimed to achieve 100% health service coverage, yet immunization coverage remains suboptimal [13]. Despite significant progress, Ethiopia still grapples with one in sixteen children not reaching the age of five, with 19% of children under 5 not being immunized [14]. This contributes to approximately 29,000 children worldwide dying each day from vaccine-preventable diseases, with Ethiopia alone witnessing around 470,000 child deaths annually [15]. This alarming scenario, with a 30-fold higher probability of death in Ethiopia compared to Western Europe, underscores the urgent need to address caregivers’ poor knowledge levels and misconceptions about child vaccination [15].

Given these pressing issues, this study aimed to examine caregivers’ perceptions about child vaccination and associated determinants in Tehuledere Woreda, South Wollo Zone, Ethiopia. Specifically, the study sought to: (1) assess caregivers’ perceptions towards child vaccination; (2) identify determinants influencing incomplete vaccination (as an indicator of vaccine hesitancy); and (3) assess rumors about child vaccinations circulating in Tehuledere Woreda. Recognizing the need for context-specific insights in every vaccination campaign, this operational research intends to provide valuable information to enhance vaccination strategies and improve immunization coverage in the region.

## Methods

### Study area and period

The research took place in Tehuledere Woreda, situated in the northeastern part of Ethiopia, within the South Wollo Zone of the Amhara Regional State. Tehuledere Woreda is one of the 20 Woredas in the South Wollo Zone of the Amhara Regional State. According to the 2007 national census conducted by the Central Statistical Agency of Ethiopia (CSA), Tehuledere Woreda has a total population of 122,288, residing in 21 kebele administrations, with 86.9% classified as rural inhabitants. The study was conducted from September 1, 2019, to November 30, 2019. *Tehuledere* Woreda is equipped with essential healthcare facilities, comprising 1 hospital, 5 health centers, and 39 health posts. Child vaccination services are available in the hospital, all health centers, and specifically in 18 health posts. Each kebele within the Woreda has health extension workers assigned to serve the community, contributing to the delivery of vital health services.

### Study design

An explanatory sequential mixed method was employed, consisting of two phases. The first phase utilized a community-based cross-sectional study design, employing a structured questionnaire in face-to-face interviews with caregivers to collect quantitative data. This was followed by a phenomenological study design in the second phase, exploring in-depth the community’s perceptions of child vaccination and rumors circulating among key informants.

### Source and study population

- **Source Population**: All households residing in Tehuledere Woreda.
- **Study Population**: Households with children aged between 12 and 36 months old in Tehuledere Woreda.
- The age range of 12–36 months was selected because the Ethiopian Expanded Programme on Immunization (EPI) schedule recommends completion of the basic vaccination series (BCG, three doses each of OPV and pentavalent vaccine, PCV, rotavirus, and measles) by 12 months of age. Extending the upper limit to 36 months allowed us to capture children who experienced delayed vaccination completion, defaulters who later returned for catch-up doses, and to assess cumulative vaccination status over a longer observation period in this rural setting where access to services may be intermittent.

### Inclusion and exclusion criteria

- **Inclusion Criteria**: Caregivers with children aged between 12 and 36 months old, available during data collection, and willing to participate in the survey.
- **Exclusion Criteria**: Caregivers not at home after repeated visits and those unwilling to participate.

### Study variables

- **Dependent/Response Variable**: Vaccination status of children aged 12–36 months. dichotomized as ‘complete’ (received BCG, three doses each of DPT and polio vaccines, and measles vaccine by 12 months of age, per Ethiopian EPI schedule) and ‘incomplete’ (missed at least one dose of the above vaccines). Incomplete vaccination is used as an operational indicator of vaccine hesitancy, consistent with the WHO SAGE definition [4].
- **Independent/Explanatory Variables**: Various socio-demographic factors, child characteristics **(including child age in months as a continuous variable)**, maternal health care utilization, and access and quality of vaccination service, and knowledge and perception about vaccination (including source of vaccination information, perceived benefit of vaccinating the child, ability to mention vaccine-preventable diseases, knowledge of age to begin and complete vaccination, and knowledge of number of sessions needed).

### Sample size determination

For the quantitative part of the study, the sample size was determined using the formula for single population proportion. Since there is no similar study done in the area, the prevalence rate was taken as 50% (0.5), with a confidence interval of 95% and a margin of error of 5% (0.05), resulting in a calculated sample size of 384. To account for the design effect due to the two-stage sampling procedure, two times the calculated sample size was taken, making the total sample size 768. Including 10% (77) compensation for the non-response rate, the total sample size reached 845.

### Sampling procedure

The sample was chosen using a two-stage sampling technique. The primary sampling units comprised 21 kebeles within the study district. Subsequently, seven rural kebeles were randomly selected from the total using a simple random sampling (lottery method). The final sample size of 845 was proportionally allocated to each of the seven selected kebeles based on their respective number of households (Table 1).

**Table 1:** Proportional allocation of sample across selected kebeles

Name of rural kebele	Total eligible households	Proportion selected (%)	Sample size
Amumo	1,450	7.9	114
Jari	1,350	7.9	106
Bededo	1,640	7.9	129
Gobeya	1,480	7.8	116
Hara	1,830	7.9	144
Kete	1,590	7.9	125
Ardibo	1,410	7.9	111
Total	**10**,**750**	**7.9**	**845**

The sampling unit was the caregiver-child pair. In households with two or more eligible children (aged 12–36 months), only one child was selected using simple random sampling (writing the names or identifiers of eligible children on separate pieces of paper, folding them identically, placing them in a container, and drawing one without replacement).

A list of eligible households (those with children aged 12–36 months) was obtained from health extension workers in each selected kebele and served as the sampling frame. Within each kebele, the sampling interval (k) was calculated as the total number of eligible households divided by the proportionally allocated sample size for that kebele. The first household was selected randomly by generating a random number between 1 and k using a random number table. Subsequent households were selected by adding the sampling interval (k) to the previous selection number. This systematic random sampling method was repeated until the proportionally allocated sample size for each kebele was achieved.

For the qualitative component of the study, community leaders considered knowledgeable about the community and capable of effectively conveying information were purposively selected by kebele managers, chairpersons, and health extension workers to conduct in-depth interviews. Each participant’s interview was recorded using an audio recorder. Data collection through these interviews continued until the point of data saturation was reached, indicating little or no new or relevant data or themes were being generated.

### Data collection tools and procedures

Data collection employed a structured questionnaire, adapted and refined from the Ethiopian EPI survey 2012 (EPI coverage survey 2012, MOH) and analogous studies in the Minjar-Shenkora district (Mekonnen et al., 2019) and Kenya (Maina et al., 2013). This questionnaire encompassed inquiries related to:

- **Socio-demographic Characteristics**: Including age, sex, formal education level, marital status, and household size of caregivers.
- **Child Characteristics**: Covering age, sex, place of delivery, preceding birth interval, and birth order.
- **Maternal Health Care Utilization**: Encompassing the use and frequency of ANC, utilization of contraception, and health-seeking behavior.
- **Access and Quality of Vaccine Service**: Including aspects such as the availability and type of nearby health institutions, means of transport, walking time, and health extension support.
- **Knowledge and Perception About Vaccination**

Quantitative data collection was executed by three pharmacy professionals after a comprehensive half-day training session. The principal investigator oversaw the coordination of quantitative data collection and conducted daily verification of completed questionnaires.

For the qualitative segment, an open-ended interview guide was meticulously developed to delve into the nuanced perceptions of the Tehuledere community regarding child vaccination and prevalent rumors. The interview guide underwent a meticulous language transformation: forward translation from English to Amharic was performed by a bilingual language expert with a Master’s degree in linguistics. A second independent translator (a health officer fluent in both languages) back-translated the Amharic version to English without reference to the original English version. The original English and back-translated English versions were compared by the research team. Discrepancies were discussed and resolved through consensus, with modifications made to the Amharic version as needed. The final Amharic version was used for data collection.

The principal investigator conducted in-depth interviews, averaging 11 min each, until thematic saturation was achieved. These interviews transpired in participants’ private residences or public spaces within villages. The interviews were conducted in Amharic, with field notes extensively used to capture the principal investigator’s experiences and observations. All interviews were recorded using an audio recorder.

### Data processing and analysis

Following the survey, completed questionnaires were submitted to the principal investigator. Quantitative data underwent coding and entry using EpiData Entry client (v4.6.0), followed by analysis with SPSS version 20 for Windows. Descriptive statistics, including frequencies and percentages, were used. Subsequently, bivariable analysis was done to test associations between independent and outcome variables. All explanatory variables associated with the outcome variable in bivariable analyses (p < = 0.25) were incorporated into multivariable logistic regression analysis to identify factors significantly associated with the dependent variable. Adjusted odds ratios (AOR) with 95% confidence intervals (CI) were reported. Statistical significance was considered at *p* < 0.05 (two-tailed).

Model assumptions were tested as follows: (1) absence of multicollinearity was assessed using variance inflation factor (VIF), with VIF < 5 considered acceptable; (2) linearity of continuous variables with the logit was assessed using the Box-Tidwell test; (3) model fit was evaluated using the Hosmer-Lemeshow goodness-of-fit test, with *p* > 0.05 indicating adequate fit; (4) overall model performance was assessed using Nagelkerke R².

Qualitative data underwent analysis using content analysis principles. Recorded interviews were transcribed verbatim in Amharic, then translated to English by a language expert. Transcriptions were carefully read and double checked for accuracy. Codes were organized into themes using an inductive, data-driven approach. Thematic saturation was assessed by reviewing transcripts sequentially.

### Data quality assurance

To ensure data quality, pretest was conducted on 5% of the total study population (*n* = 42) in a non-selected kebele (Gashana) of Tehuledere Woreda, which was not included in the final study, two weeks prior to actual fieldwork (August 15–20, 2019). The purposes of the pretest were to: (1) assess the clarity and comprehensibility of the questionnaire; (2) estimate the average time required for interview completion; (3) identify ambiguous, confusing, or culturally inappropriate questions; and (4) test the data entry template. Based on pretest findings, minor wording modifications were made to three questions (specifically, clarifying the definition of ‘fully vaccinated’ and simplifying response options for ‘source of vaccination information’). No major structural changes were required. All collected data underwent rigorous examination for completeness, accuracy, clarity, and consistency throughout data collection, analysis, and interpretation. During data collection, supervisors were checking the data collection process. At the end of each collection day, the principal investigator and supervisors checked the completeness of filled questionnaires. For binary logistic regression, the following assumptions were tested and met: (1) independence of observations (confirmed by sampling design—one child per household); (2) absence of multicollinearity (VIF ranged from 1.1 to 2.8, all < 5); (3) linearity of continuous variables with the logit (Box-Tidwell test *p* > 0.05 for child age in months); (4) no influential outliers (Cook’s distance values all < 1). Model fit was confirmed using the Hosmer-Lemeshow test (*p* = 0.987, see Annex V in supplementary file).

The in-depth interview guide was prepared in English and back-translated into Amharic to check message consistency. The Amharic version was used for interviews with key informants. The Amharic transcript was returned to key informants and signed. Transcriptions were meticulously reviewed for accuracy by a language expert.

### Issue of reflexivity

The principal investigator’s status as a “professional” offered certain advantages and limitations for this study. The study was conducted in the area where the researcher had become socialized. He was also faced with the challenge of being perceived as a powerful individual based on his position as a member of community elite and a senior clinical pharmacist. Careful consideration was given to the identity of the researcher and the potential power imbalance in the interview setting. Reflexivity was employed to ensure that his personal opinions remained undisclosed to the participants. He did not interfere with or alter the opinions of the participants. He made the interview open-ended without any set agenda and he documented every data through the lens of the participants. Furthermore, audio recordings and verbatim translations greatly reduced possible inherent biases.

### Operational definitions

**Key operational definitions are provided here; the complete list is available in Additional File** 1 **.**

- **Fully Vaccinated**: children are considered as fully vaccinated when they have received a vaccination against tuberculosis (BCG), three doses each of the DPT and polio vaccines and measles vaccination by the age of 12 months.
- **Partially Vaccinated**: children are considered as partially vaccinated when they miss at least one dose of the above mentioned vaccines on fully vaccinated definition.
- **Unvaccinated**: children are considered as unvaccinated when they did not receive any dose of the above mentioned vaccines on fully vaccinated definition.
- **Vaccine Hesitancy**: a delay in acceptance or refusal of vaccination despite availability of vaccination services [4] (Larson et al. 2015).

The complete list of operational definitions, including definitions for caregiver, community, child vaccination, vaccinated, health service utilization of mothers, and vaccine, is provided in Additional File 1 (Supplementary Material).

### Ethical consideration

The research proposal received approval from the Ethics Review Committee of the College of Medicine and Health Sciences, Wollo University (approval number: CHMS/408/13/11) on August 15, 2019, and Tehuledere Woreda Administration Office (reference number: /429/2012) on August 22, 2019** before data collection. All procedures were conducted in accordance with the Declaration of Helsinki. Participants were informed about the study’s purpose, the reasons for their selection, and what was expected of them. Confidentiality of information throughout the study was assured, and the names of households were coded in the data collection format.

## Results

### Socio-demographic characteristics of respondents

A total of 824 caregivers of children aged 12 to 36 months participated in the survey, yielding a response rate of 97.5%. The majority of caregivers (52.4%) were aged 31 to 40 years. Regarding marital status, 74.4% were currently married, 13.5% were divorced or separated, 9.7% were widowed, and 2.7% were single. The predominant religion among caregivers was Islam, with 78.4% identifying as Muslim and 21.6% as Christian (Table 2).

**Table 2:** Socio-demographic characteristics of caregivers (*n* = 824)

Variable	Category	Frequency (*N*)	Percentage (%)
Age	20–30 years	245	29.7
	31–40 years	432	52.4
	41–50 years	147	17.8
Level of formal education	No formal education	290	35.2
	Primary education	397	48.2
	Secondary education	137	16.6
Religion	Christianity	178	21.6
	Muslim	646	78.4
Marital status	Married	613	74.4
	Single	20	2.4
	Divorced/separated	111	13.5
	Widowed	80	9.7
Household size	≤ 4	481	58.4
	≥ 5	343	41.6

### Characteristics of the children

The age distribution of children revealed that 45.4% were between 19 and 24 months old, 21.6% were between 25 and 30 months, and 20.8% were between 12 and 18 months. A total of 53.3% of the children were female, and 46.7% were male. The majority of children (64.2%) were born more than 37 months after their preceding sibling, and 77.2% were born at health facilities (Table 3).

**Table 3:** Characteristics of the children (*n* = 824)

Variable	Category	Frequency (*N*)	Percentage (%)
Age in months	12–18	171	20.8
	19–24	374	45.4
	25–30	178	21.6
	31–36	101	12.3
Sex	Male	385	46.7
	Female	439	53.3
Place of delivery	Home	188	22.8
	Health facility	636	77.2
Preceding birth interval	≤ 24 months	176	21.4
	25–36 months	119	14.4
	≥ 37 months	529	64.2
Birth order	1	35	4.2
	2	296	35.9
	3	256	31.1
	4	189	22.9
	5	48	5.8
Occurrence of child death in family	Yes	95	11.5
	No	729	88.5

### Knowledge on immunization and vaccine preventable diseases

The primary sources of vaccination information for caregivers were community members (30.8%) and health extension workers (21.6%). Most caregivers (59.1%) believed vaccination prevents disease, whereas 15.3% thought it treated childhood diseases. Caregivers who could identify at least two vaccine-preventable diseases were 36.7%, and those who could not name any were 19.7%. The majority of caregivers (50.6%) believed that vaccination begins at 6 weeks, while 23.4% thought it starts at 4 weeks. Completion of vaccination was most commonly reported as being at 9 months (55%), and 16% did not know the completion age (Table 4).

**Table 4:** Caregivers’ knowledge on vaccination and vaccine-preventable diseases (*n* = 824)

Variable	Category	Frequency (*N*)	Percentage (%)
Source of vaccine information	Community members	254	30.8
	Health workers at health facility	156	18.9
	Health extension workers	178	21.6
	Mass media	103	12.5
	Kebele administrator	133	16.1
Benefit of vaccinating child	Treating childhood disease	126	15.3
	Preventing childhood disease	487	59.1
	For specific disease	84	10.2
	I don’t know	127	15.4
Mentioned vaccine-preventable diseases	≤ 2	302	36.7
	3–4	208	25.2
	≥ 5	152	18.4
	I don’t know	162	19.7
Vaccination may cause health problem	Yes	112	13.6
	No	712	86.4
Age to begin vaccination	At birth	167	20.3
	4 weeks	193	23.4
	6 weeks	417	50.6
	I don’t know	47	5.7
Vaccine sessions to complete	3 sessions	34	4.1
	4 sessions	319	38.7
	5 sessions	307	37.3
	I don’t know	164	19.9
Age to complete vaccination	9 months	453	55.0
	12 months	176	21.4
	15 months	42	5.1
	I don’t know	132	16.0

### Maternal health care utilization, access and quality of immunization services

Among respondents, 84.8% attended antenatal care (ANC), with 70.5% attending 3 or more times. Most (75.5%) delivered their last child at a health facility. Utilization of modern contraceptives was reported by 66.7%, and 67.5% sought health facility care for their children.

In the study area, most caregivers (66.9%) reported that vaccinations were administered at health posts, while 26% used health centers, and 7.2% attended hospitals. Accessibility to vaccination sites varies: the majority of caregivers (86.7%) walk to the facilities, whereas 13.3% use transport. Walking times to the nearest health facility are generally moderate, with 51.3% walking for 15–30 min, 29.6% taking less than 15 min, and 19.1% walking for over 30 min.

Regarding health extension workers (HEWs), 520 (63.1%) receive regular visits and advice, while 304 (36.9%) do not. On vaccination days, 677 (82.2%) did not experience long waiting lines, and 595 (72.2%) rated the service as good. Only 123 (14.9%) were refused service, mainly due to vaccine shortages (65, 52.9%), missed vaccination days (19, 15.4%), or illness (21, 17.1%) (Table 5).

**Table 5:** Maternal health care utilization and service access (*n* = 824)

Variable	Category	Frequency (*N*)	Percentage (%)
Mother attended ANC	Yes	699	84.8
	No	125	15.2
Frequency of ANC	≤ 2 times	206	29.5
	≥ 3 times	493	70.5
Place of delivery	Home delivery	202	24.5
	Health facility delivery	622	75.5
Health seeking place for child	Self-treatment at home	170	20.6
	Health facility	556	67.5
	Other place	98	11.9
Utilization of modern contraceptive	Yes	550	66.7
	No	274	33.3
Type of nearby health facility	Health center	214	26.0
	Hospital	59	7.2
	Health post	551	66.9
Means of transport to facility	Walk	714	86.7
	By transport	110	13.3
Walking time to facility	< 15 min	244	29.6
	15–30 min	423	51.3
	30 min–1 h	157	19.1
HEWs perform home visit	Yes	520	63.1
	No	304	36.9
HEWs give advice about vaccination	Yes	526	63.9
	No	298	36.2
Presence of long waiting line	Yes	147	17.8
	No	677	82.2
Satisfaction with vaccination service	Good	595	72.2
	Medium	229	27.8
Refused vaccination service	Yes	123	14.9
	No	701	85.1

### Vaccination status

Among the 824 children, 775 (94.1%) received at least one vaccine, while 49 (5.9%) received none. Of those surveyed, 598 (77.2%) had vaccination cards at home. Fully vaccinated children aged 12–36 months were 596 (72.3%) by recall and 504 (61.1%) by card. **For the logistic regression analysis**,** vaccination status was determined by combining both sources: a child was classified as fully vaccinated if either the vaccination card OR maternal recall indicated completion of all required vaccines. This combined classification served as the dependent variable in the regression models.**

### Reasons for defaulting vaccination

For the 179 children who were partially vaccinated, the most common reasons for defaulting were inconvenient vaccination times (41%), lack of awareness about vaccination schedules (28%), perceived lack of importance (12%), fear of side effects (12%), and distance to vaccination sites (6%).

### Determinants of vaccination status

An analysis of factors influencing vaccination status considered socio-demographics, child characteristics, maternal health care utilization, and service quality.

#### Socio-demographic and knowledge-related factors

Caregivers’ marital status and educational level, along with household size, significantly influenced vaccination status. Specifically, widowed caregivers were 7.3 times more likely to have incompletely vaccinated children (AOR = 7.3, 95% CI: 2.2–24.5), and divorced caregivers were 4 times more likely (AOR = 4.0, 95% CI: 1.8–14.8). Those without formal education were 2.2 times more likely to have incomplete vaccination (AOR = 2.2, 95% CI: 1.2–3.6). Larger household sizes (≥ 5 members) were associated with a 5.4 times higher likelihood of incomplete vaccination (AOR = 5.4, 95% CI: 3.2–9.3).

Caregivers who received vaccination information from community members were 4.2 times more likely to have incompletely vaccinated children (AOR = 4.2, 95% CI: 1.7–9.6). Those who did not understand the purpose of vaccination were 11.5 times more likely to have incomplete vaccination (AOR = 11.6, 95% CI: 5.5–24.6). Caregivers who could not name any vaccine-preventable diseases were 3.6 times more likely to have incomplete vaccination (AOR = 3.6, 95% CI: 1.8–7.3). (Table 6).

**Table 6:** Socio-demographic and knowledge-related factors associated with incomplete vaccination (*n* = 824)

Variable	Category	Incomplete (*n*)	Complete (*n*)	COR (95% CI)	AOR (95% CI)	*p*-value
Marital status	Married (Ref)	53	560	1	1	
	Single	8	12	3.0 (1.2–7.6)	-	-
	Divorced/separated	93	18	5.3 (2.9–9.6)	4.0 (1.8–14.8)	< 0.001
	Widowed	76	4	12.6 (4.3–36.8)	7.3 (2.2–24.5)	0.001
Household size	≤ 4 (Ref)	68	413	1.2 (0.9–1.7)	1	
	≥ 5	160	183	6.0 (4.1–8.8)	5.4 (3.2–9.3)	0.0012
Education	No formal	91	199	4.7 (3.0-7.4)	2.2 (1.2–3.6)	< 0.001
	Primary	69	328	1.0 (0.7–1.5)	-	-
	Secondary (Ref)	68	69	1	1	
Source of information	Community members	131	123	3.2 (1.8–5.7)	4.2 (1.7–9.6)	0.001
	Health workers at facility	29	127	1.2 (0.6–2.3)	1.8 (0.9–3.6)	
	Health extension workers (Ref)	17	161	1	1	
	Mass media	20	83	1.2 (0.6–2.4)	1.9 (0.9–4.1)	
	Kebele administrator	31	102	2.8 (1.4–5.6)	2.5 (1.07–5.8)	
Benefit of vaccination	Preventing disease (Ref)	61	426	1	1	
	Treating disease	20	106	3.0 (1.7–5.3)	1.7 (0.8–3.4)	
	For specific disease	44	40	29.0 (15.2–55.3)	10.6 (5.2–20.8)	
	I don’t know	103	24	22.0 (11.5–42.1)	11.6 (5.5–24.6)	< 0.001
Mention VPDs	≥ 5 (Ref)	1	151	1	1	
	≤ 2	95	207	13.3 (7.5–23.6)	2.2 (1.0-3.8)	0.002
	3–4	26	152	2.8 (1.5–5.2)	-	-
	I don’t know	106	56	4.3 (2.4–7.6)	3.6 (1.8–7.3)	0.012
Vaccine sessions to complete	5 (Ref)	34	273	1	1	
	3	12	22	5.0 (2.1–11.9)	2.2 (1.2–4.3)	
	4	76	243	14.0 (6.8–28.8)	1.2 (0.4–3.3)	
	I don’t know	106	58	3.4 (2.0-5.8)	2.6 (1.4-5.0)	0.005

Footnote: p-values derived from Wald test in multivariable logistic regression. Dependent variable: incomplete vaccination (reference: complete). Model fit: Hosmer-Lemeshow *p* = 0.987

#### Child characteristics

Children born at home were more likely to be incompletely vaccinated (OR 4.6, 95% CI 1.8–10). Those who experienced child death in the family were also more likely to be incomplete (OR 2.6, 95% CI 1.2–6.8). **Child age in months was not significantly associated with incomplete vaccination (AOR = 1.02**, **95% CI: 0.99–1.05**, ***p = 0.142)*** (Table 7).

**Table 7:** Child characteristics associated with incomplete vaccination (*n* = 824)

Variable	Category	Incomplete (*n*)	Complete (*n*)	COR (95% CI)	AOR (95% CI)	*p*-value
Child age (months)	Continuous	Mean ± SD: 24.6 ± 7.2	Mean ± SD: 22.8 ± 6.9	1.03 (1.01–1.06)	1.02 (0.99–1.05)	0.142
Place of delivery	Home	155	40	7.6 (4.8–12.0)	4.6 (1.8–10.0)	0.0012
	Health facility (Ref)	73	556	1	1	
Preceding birth interval	≤ 24 months	152	24	12.8 (7.6–21.5)	4.8 (2.4–12.6)	0.002
	25–36 months	42	77	1.3 (0.8–2.1)	-	-
	≥ 37 months (Ref)	82	447	1	1	
Child death in family	Yes	20	4	5.2 (1.6–16.8)	2.6 (1.2–6.8)	0.004
	No (Ref)	53	345	1	1	

Footnote: p-values derived from Wald test in multivariable logistic regression. Dependent variable: incomplete vaccination (reference: complete)

#### Maternal health care utilization, access and quality factors

The study analyzed the relationship between maternal health care utilization and child vaccination status. It found that mothers who attended antenatal care (ANC) two times or fewer were 2.1 times more likely to have incompletely vaccinated children (AOR = 2.1, 95% CI: 1.09–4.4) compared to those who attended ANC three times or more. Additionally, mothers not using modern contraceptives were 10 times more likely (AOR = 10.0, 95% CI: 5.2–20.4) to have incompletely vaccinated than those who did use modern methods. The study also highlighted that mothers who relied on home treatment for childhood illnesses were 2.5 times more likely (AOR = 2.5, 95% CI: 1.15–5.4) to have incompletely vaccinated children compared to those who sought care at health facilities.

This section also examined how access to and quality of vaccination services impacted vaccination completion. It was found that although all children had access to health facilities, the type of facility mattered: caregivers who used health posts were 4.07 times more likely to have incompletely vaccinated children (AOR = 4.1, 95% CI: 1.6–9.8) compared to those using hospitals. The involvement of health extension workers (HEWs) also played a significant role; caregivers who did not receive home visits from HEWs were 14.7 times more likely to have incomplete vaccinated childeren (AOR = 14.7, 95% CI: 8.2–26.4). Furthermore, the time it took to reach a health facility was a significant factor, with those taking over 30 min being 9.5 times more likely higher likelihood of incomplete vaccination (AOR = 9.5, 95% CI: 3.2–27.9) compared to those who took less than 15 min (Table 8).

**Table 8:** Maternal health care, access and quality factors associated with incomplete vaccination (*n* = 824)

Variable	Category	Incomplete (*n*)	Complete (*n*)	AOR (95% CI)	*p*-value
Frequency of ANC	≤ 2 times	72	134	2.1 (1.09–4.4)	0.0012
	≥ 3 times (Ref)	70	423	1	
Health seeking place	Home treatment	117	53	2.5 (1.15–5.4)	< 0.001
	Health facility (Ref)	39	517	1	
Contraceptive use	No	185	89	10.0 (5.2–20.4)	< 0.001
	Yes (Ref)	89	507	1	
Type of facility	Hospital (Ref)	3	56	1	
	Health center	13	201	1.5 (0.4–9.8)	
	Health post	212	339	4.1 (1.6–9.8)	0.002
HEW home visit	Yes (Ref)	26	494	1	
	No	202	102	14.7 (8.2–26.4)	0.002
Walking time	< 15 min (Ref)	13	231	1	
	15–30 min	63	360	2.8 (0.7–10.9)	
	> 30 min	152	5	9.5 (3.2–27.9)	< 0.001

Footnote: p-values derived from Wald test in multivariable logistic regression. Dependent variable: incomplete vaccination (reference: complete)

### Qualitative findings

#### Demographic characteristics of participants in qualitative study

A total of 24 participants from the community leaders of each kebele were selected for in-depth interviews. Participants were within the ages of 46 to 65, with a mean age of 56.34 ± 5.51 years. Among the participants, 3 (12.5%) were females, and 21 (87.5%) were males, including 3 religious leaders. All names credited to the quotes below are pseudonyms.

#### Perception towards child vaccination

All the study participants were asked to articulate distinctly their lived experiences regarding how the community members understand and interpret child vaccination. They were also asked to discuss the common rumors circulating among the communities of Tehuledere Woreda.

One of the findings was the importance of childhood vaccination. According to the interviewees’ responses, controversial perceptions were found among the community of Tehuledere Woreda towards child vaccination.

The majority of the participants (*n* = 14) indicated that caregivers of children understand the importance of vaccines for their children. They view vaccination as a way of protecting children from deadly childhood epidemics and ensuring healthy development. Religious and community leaders play a crucial role by providing information, encouraging vaccination, and engaging in dialogue across multiple levels of the Tehuledere community to influence members positively towards child vaccination, thus increasing vaccination rates and reducing vaccine hesitancy.

#### Sheik Siraj, a 62-year-old religious leader, stated

We are comfortable with child vaccination practice and people usually take their children to the vaccination site. We consider vaccination as medicine, and it makes the children grow fast

#### Another 58-year-old male participant said

The community is pleased about child vaccination and keeps up with the vaccination program as the imams (religious leaders) and the EDIR (community-based organization) leaders, along with community health workers, advocate the program.

#### Zelalem, a 56-year-old participant, shared

Child vaccination is a very good activity as many diseases, which were a social problem when we were kids, are now gone. I have three children, two of whom have completed their vaccinations, and one is currently being vaccinated. I saw the villagers remember the vaccination days because they believe vaccinating children is like making a strong fence around their home and their garden.

Another perception found in the community is that vaccination increases children’s mental development. According to the participants, some community members believe that child vaccination not only ensures proper growth and health but also enhances intelligence and social participation. This perception is shaped by the perceived benefits of vaccines.

#### Zewudu, a 51-year-old participant, said

Our community members used to vaccinate their children regularly. I also had my daughter vaccinated because child vaccination made children intelligent, healthy, and well-developed.

#### Another 58-year-old male participant stated

I have four kids; some of the elders didn’t get vaccinated, but the younger ones are vaccinated. There is a marked difference between vaccinated and non-vaccinated children in terms of their strength and mental ability.

Some participants (*n* = 4) indicated that child vaccination might cause health problems. Participants revealed that whenever their children experienced health issues during vaccination, they attributed the problems to the vaccination without seeking clinical confirmation, affecting their decision to vaccinate subsequent children.

#### Lubaba, a 55-year-old female participant, stated

My elder son was vaccinated, but the vaccine caused his body to swell badly. For my next child, I hid my baby to avoid vaccination because I worried the same event might occur.

Another female participant indicated that it is unnecessary to vaccinate children if they become ill in infancy. She explained: “I didn’t vaccinate my son because, at an early age, he suffered from ‘AYNETILA’ (a recurring childhood disease). Vaccination and AYNETILA are very unfriendly.”

Religious beliefs and cultural norms also influence perceptions towards child vaccination.

#### Zeyneba, a 52-year-old Muslim female participant, said

We believe in Allah. We believe also that all harms and benefits are from Allah. Nothing can prevent us from harm if Allah decides it. There is no difference for my baby whether I vaccinate him or not. But I took my baby to be vaccinated following health extension workers’ promotion and call.

#### Fentaw, a 64-year-old male participant, stated

When very young children are exposed to be seen by others, especially in public places, they will develop discomfort and become unhealthy. People from different ‘AHWAL’ (states of mood) gather at vaccination sites, making these sites dangerous places for children.

### Rumors towards child vaccination

Despite the recognized importance of vaccination, persistent rumors and misconceptions contribute to incomplete vaccinetion and lower immunization rates in the community. Some rumors include the belief that vaccines are expired or useless drugs, part of a secret hostile plan, or an act of political revenge. These beliefs suggest that vaccination affects children’s natural growth, physical strength, eyesight, and reproductive power.

#### Ali, a 57-year-old male participant, said

I saw individuals who believe and disseminate their beliefs that vaccination is a way of drug disposal. The government sends medicines that are expired, cheap, and useless in the name of child vaccination when other places are not convenient.

#### Another 48-year-old male participant stated

Formerly, our people believed that child vaccination is a conspiracy of others. This belief was that vaccination makes our children fat and grow fast; once the children grew and became fat, the vaccinators would take and sell them to another place.

#### Zegeye, a 48-year-old male participant, shared

At the time of political tensions, a rumor circulated among the public that child vaccination is a conspiracy by the enemy to make Amhara kids stunted, less strong, and weak-sighted.

#### Another 54-year-old male participant also said

It is said, and we suspect, that the so-called child vaccination may harm our children. Despite its validity to protect them from injury, it might be an enemy conspiracy to bring long-term effects on children’s proper growth, health, physical strength, and even their reproductive power.

#### Tefera, a 54-year-old male participant, said

We heard disturbing information from some individuals. It is said that vaccines contain chemicals with different functions. One nourishes the child for good growth and development, but the other is very dangerous. Early vaccinated children will be disloyal to their families and will go against their country’s promise when they become adults.

## Discussion

This study, conducted in the rural community of Tehuledere Woreda, assessed caregivers’ perceptions towards child vaccination and factors contributing to incomplete vaccination among children aged 12 to 36 months.

Using vaccination cards and maternal recall, we found that 596 children (72.3%) were fully vaccinated according to the vaccination cards, and 504 children (61.2%) were fully vaccinated based on maternal recall. These findings are consistent with studies conducted in Minjar Shenkora Woreda [16] and Nigeria [17], but higher than studies from Mizan Woreda [18] and Zimbabwe (Elia et al., 2016). Conversely, the vaccination rates were lower than those found in Sina District [19] in Southern Ethiopia.

Our study found a significant association between family size and vaccination completion, with larger families more likely to have incomplete vaccinations. This is consistent with findings from Kenya [20], suggesting that resource dilution in larger families limits access to necessary healthcare, including vaccinations.

Marital status was another significant factor; children of widowed caregivers were more likely to have incomplete vaccinations compared to those with married caregivers, aligning with findings from Minjar Shenkora Woreda [16]. The economic and time constraints faced by widowed mothers might explain this association.

Caregivers who received vaccination information from community members were more likely to have incomplete vaccinations compared to those who received information from health extension workers. Community sources may spread misinformation, leading to incomplete vaccinations. This finding underscores the critical role of trained health professionals in vaccine communication.

Educational level significantly influenced vaccination status; caregivers without formal education were more likely to have incomplete vaccinations than those with secondary education. This is supported by studies from Sinana District, Libya, Malaysia, and Pakistan [19, 21–23]. Educated caregivers likely have better access to accurate health information.

Maternal healthcare utilization, specifically ANC attendance and delivery place, showed significant associations. Children born at home were more likely to have incomplete vaccinations compared to those born in health facilities, consistent with findings from Matakel Woreda, Nigeria, and Bangladesh [24–26]. This association, however, was not found in a Malaysian study [27]. Health facility delivery serves as an entry point to the formal health system, where mothers receive vaccination cards and initial guidance.

Child death in a family was also associated with vaccination status, with caregivers who experienced child death more likely to have incomplete vaccinations. This may stem from the fear of another death among their children or potentially from prior negative healthcare experiences.

Caregivers’ health-seeking behavior was another significant factor; those who used homemade treatments were more likely to have incomplete vaccinations compared to those who sought medical help from health facilities. Preference for traditional medicine over allopathic medicine impacts vaccination decisions.

Caregivers who did not use modern contraceptives were more likely to have incomplete vaccinations compared to those who did, possibly due to less frequent contact with health professionals among non-users.

Knowledge about the immunization timetable and vaccine-preventable diseases was crucial. Caregivers unaware of the correct number of sessions needed for complete vaccination were more likely to have incomplete vaccinations compared to those who knew 5 sessions were needed. This is consistent with studies from Debre Markos and Ambo [28, 29].

Health care availability and accessibility significantly impacted vaccination status. Caregivers receiving services from health posts were more likely to have incomplete vaccinations compared to those receiving services from hospitals. This may be due to the higher qualifications and better counseling provided by hospital staff. Travel time to health facilities also mattered; caregivers taking more than 30 min to reach a health facility were more likely to have incomplete vaccinations, aligning with findings from Sinana Woreda [19].

The competence of health extension workers was significant. Caregivers not regularly visited by health extension workers were more likely to have incomplete vaccinations, consistent with studies from Machakel Woreda and Bangladesh [25, 26]. Health extension workers play a critical role in informing, sensitizing, and mobilizing the community, ensuring routine vaccination is well-accepted and known to be essential for child health.

The in-depth interview results revealed that religious influence and cultural norms contribute to incomplete vaccinations, consistent with studies from Sinana and Mizan Ambo woredas [19, 21]. Despite government efforts, misinformation and negative perceptions persist, similar to findings in Nigeria [26].

## Strengths and limitations of the study

### Strengths

The high response rate (97.5%) minimizes the risk of non-response bias and enhances the representativeness of our findings for the study population. The mixed-methods approach allowed triangulation of quantitative findings with qualitative insights from community leaders, providing a comprehensive understanding of vaccination perceptions. The use of both vaccination cards and maternal recall to determine vaccination status reduced misclassification compared to recall-only approaches. The insider positionality of the principal investigator, being familiar with the local language, culture, and social norms—facilitated participant rapport and reduced potential communication barriers during in-depth interviews. Use of mixed method approach was included to answer and clarify community perception issues on the study.

### Limitations

Several limitations should be acknowledged. First, the cross-sectional study design precludes establishing causal relationships between identified factors and vaccination status; only associations can be reported. Second, despite efforts to verify vaccination status using cards, maternal recall bias may have occurred, particularly for children aged 24–36 months whose early vaccinations were more distant. Third, social desirability bias may have influenced participants’ responses during interviews, potentially overestimating reported vaccination acceptance. Fourth, the insider positionality of the principal investigator, while facilitating access and rapport, could introduce bias if participants modified their responses based on perceived expectations; reflexivity measures, including open-ended questioning without a set agenda and verbatim transcription, were employed to mitigate this risk. Fifth, residual confounding from unmeasured variables (e.g., paternal attitudes toward vaccination, household wealth beyond monthly income, specific religious teachings, and prior experiences with vaccine adverse events) may influence the observed associations. Finally, the findings are specific to Tehuledere Woreda and may not be directly generalizable to other regions of Ethiopia or other countries with different health system structures and cultural contexts.

## Practical implication of the study

Vaccines, despite being cost-effective health investments with huge impacts on health, avoiding millions of deaths per year and increasing life expectancy, are not universally accepted. Trust in the safety and efficacy of vaccines, trust in the individuals that administer vaccines or give advice about vaccination, and trust in the wider health system are all important factors which influence the vaccine decision-making process. Recognizing trust as a complex web of vaccine-related factors can provide valuable insights into levers of vaccine acceptance, hesitancy or refusal. Effective vaccination services depend on building community trust through strong relationships between healthcare providers and community members.

The behavioral outcomes generally associated with incomplete vaccinations in Tehuledere community may reflect a decision-making process that is not guided by a general principle towards vaccination. The finding of the research is relevant for creating awareness on the influence of vaccine decision making process. Responsible stakeholders must formulate interventions to maximize vaccine acceptance. Source of health information the community utilizes is crucial. The importance of communication in strengthening knowledge and empowering actions related to uptake of modern health services and vaccination is significant. Therefore, it will be good practice considering religious and traditional leaders’ engagement and dialogue to address incomplete vaccination/refusal and improve child vaccination acceptance and at last child health outcomes. This measure also strengthens the reaching every district (RED) approach which has been implemented in Ethiopia since 2004 in districts with poor immunization coverage and high dropout rates.

## Conclusion and recommendation

Timely immunization is critical for disease prevention, yet vaccine decision-making is influenced by numerous factors. This study identified several determinants of incomplete childhood vaccination in Tehuledere Woreda, including marital status, place of delivery, family size, child mortality in the family, sources of information on vaccination, health-seeking behaviors, antenatal care attendance, knowledge about the purpose of vaccination, vaccination schedules, and vaccine-preventable diseases. The role of health extension workers, the type of health facility, and the distance to these facilities were also significant. While many participants recognized the benefits of vaccination, misconceptions persisted. Health extension workers played a crucial role in promoting regular antenatal care and institutional deliveries, which fostered trust in vaccination programs. Given that child immunization rates in the study area still fall short of the governmental target of 90% by 2020, it is essential to improve mothers’ health service utilization and provide clear information about vaccine contraindications and side effects.

### Recommendations

#### For Tehuledere Woreda health office

- Ensure continuous vaccine availability and appropriate storage.
- Expand vaccination sites to improve accessibility for distant communities.
- Scale up community health education programs.

#### For health professionals

- Increase antenatal care follow-up, family planning coverage, and institutional delivery through rigorous consultation.
- Provide clear information on vaccine contraindications and side effects.

#### For Amhara national regional state

- Consider implementing policies that mandate immunization completion certificates for school entry.

#### For researchers

- Conduct further studies to understand the perceptions of vaccine defaulters and deniers.

## Supplementary Information

Below is the link to the electronic supplementary material.

Supplementary Material 1

## Data Availability

The datasets generated during and/or analyzed during the current study are available from the corresponding author on reasonable request.
