# Supplementary material for: Perception about child vaccination and its determinants among caregivers in rural Northeastern Ethiopia
Source: Res Health Serv Reg. 2026 Aug 12;5:12. doi: 10.1007/s43999-026-00097-1 (PMC13468827; doi:10.1007/s43999-026-00097-1)
Supplement: Supplementary file 1 [file 43999_2026_97_MOESM1_ESM.docx]

**Operational Definitions**

The operational definitions employed in this study are as follows:

- **Caregivers:** Biological mothers or the individuals primarily responsible for providing care to children aged 12 to 36 months.
- **Community:** A collective of individuals residing in the same defined area, sharing common basic values, organization, and interests.
- **Child Vaccination:** The process involving the injection or introduction of a vaccine into a child to confer immunity against a specific disease.
- **Fully Vaccinated:** children are considered as fully vaccinated when they have received a vaccination against tuberculosis (BCG), three doses each of the DPT and polio vaccines and measles vaccination by the age of 12 months.
- **Partially Vaccinated:** children are considered as partially vaccinated when they miss at least one dose of the above mentioned vaccines on fully vaccinated definition.
- **Unvaccinated:** children are considered as unvaccinated when they did not receive any dose of the above mentioned vaccines on fully vaccinated definition.
- **Vaccinated:** Children are regarded as vaccinated when they have received at least one dose of the vaccines mentioned in the fully vaccinated definition.
- **Health Service Utilization of Mothers:** The utilization of antenatal care (ANC), contraceptive services, and delivery services by mothers, obtained from either government or private health institutions.
- **Vaccine:** A preparation of weakened or attenuated bacteria or viruses that can be injected to confer immunity against a specific disease.
- **Vaccine Hesitancy:** a delay in acceptance or refusal of vaccination despite availability of vaccination services [1].
